# Commentary: Dopamine-Dependent Loss Aversion during Effort-Based Decision-Making

**DOI:** 10.3389/fnins.2020.00468

**Published:** 2020-05-13

**Authors:** Russell J. Boag

**Affiliations:** Department of Psychology, University of Amsterdam, Amsterdam, Netherlands

**Keywords:** loss aversion, effort-based decision making, sequential sampling models, model-based cognitive neuroscience, Parkinson's disease

In a recent study, Chen et al. (2019) found that medicated Parkinson's patients were less loss averse than healthy controls in an effort-based decision-making task. *Loss aversion* refers to a tendency to weight losses (punishments) more heavily than equivalent gains (rewards) (Kahneman and Tversky, 1979; Tversky and Kahneman, 1992), while *effort-based decision making* refers to tasks in which responding requires the exertion of physical effort (Kurniawan et al., 2011). Chen et al. found that when decisions were framed in terms of maximizing gains, Parkinson's patients and healthy controls were equally motivated to exert effort to obtain reward. However, when framed in terms of minimizing losses, patients were less motivated to exert effort to avoid punishment. Given that many clinical disorders are characterized by aberrant motivational states (Rahman et al., 2001; Cléry-Melin et al., 2011; Baraduc et al., 2013; Chong et al., 2015) and abnormal sensitivity to rewards and punishments (Kobayakawa et al., 2010; Treadway et al., 2012), studying effort-based decision making can provide important insights into the behavioral effects of such disorders and the mechanisms by which they arise.

In Chen et al., participants performed a typical effort-based decision-making task: On each trial they were shown an amount of points and a level of physical force required to execute the decision (using an individually-calibrated dynamometer). In the reward context, participants could choose to either exert the displayed force to gain the points (obtain reward) or skip the trial and receive nothing. In the punishment context, participants could exert the force to receive nothing (avoid punishment) or skip the trial and lose the points. Trial duration was fixed to avoid confounding temporal discounting with effort discounting. To the resulting choice data, the authors fit several computational models of effort discounting, which compute choice utility based on the size of the reward/punishment attenuated by the effort required to respond (Hartmann et al., 2013). For example, Chen et al.'s (best-fitting) parabolic discounting function is given by:

(1) $$\begin{array}{r} U_{t}=R_{t}-\alpha E_{t}^{2} \end{array}$$

where *U* represents choice utility, *R* is the reward/punishment amount, *E* is the effort required, α is an effort-discounting parameter, and *t* indexes trials. The probability of choosing to exert effort on a given trial is then computed using a soft-max choice function that scales choice utility by a choice stochasticity parameter, β:

(2) $$\begin{array}{r} p{\left(effort\right)}_{t}=\frac{1}{1+e^{-\beta^{*}U_{t}}} \end{array}$$

Parameter estimates from these models supported the behavioral results: Patients and controls had similar average effort-discounting parameters in the reward context, but patients had significantly higher effort-discounting parameters in the punishment context, confirming that patients were less motivated to exert effort to avoid punishment.

Although Chen et al. did not explicitly model the latent cognitive processes driving observed choice behavior (nor did they include any neurophysiological measures), the authors speculate that their findings could be due to dopaminergic Parkinson's medication differentially suppressing activity in Basal Ganglia pathways associated with processing punishment (Frank, 2005; Argyelan et al., 2018). This suggestion is consistent with several computational reinforcement learning studies linking reduced Basal Ganglia dopamine activity with impaired learning on punishment-based tasks in medicated Parkinson's patients (Frank et al., 2004; Frank, 2005). However, without additional neurophysiological measures (e.g., fMRI, PET) and an appropriate cognitive model of latent decision processes (e.g., Ratcliff, 1978; Brown and Heathcote, 2008), it is difficult to draw strong conclusions about the latent mechanisms driving the observed loss aversion effects or their neurophysiological basis from Chen et al.'s analyses.

A natural starting point for extending Chen et al.'s modeling to answer such questions would be to augment their soft-max choice rule and utility-based effort-discounting functions with a more comprehensive cognitive process model of decision making, such as a *sequential sampling model*—the most successful and widely applied class of decision-making models in model-based cognitive neuroscience (for reviews, Mulder et al., 2014; Forstmann et al., 2016). Sequential sampling models treat decision making as a process of accumulating samples of evidence^1^ from stimuli until a threshold amount is reached, triggering a response (Figure 1).

**Figure 1:**
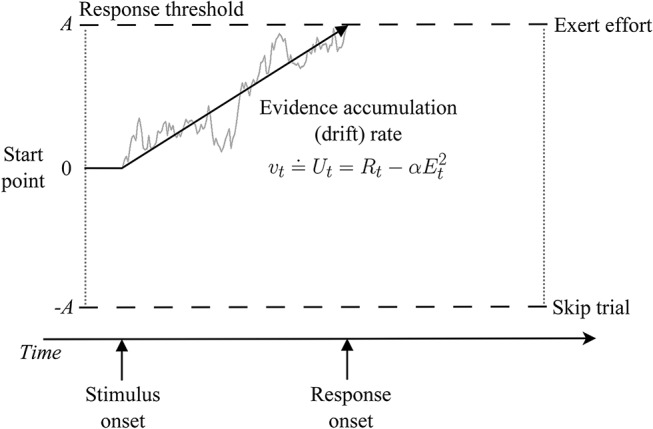
Illustration of a sequential sampling model of effort-based decision making. Within a trial, evidence about the relative value of each choice option (exert effort vs. skip trial) is accumulated over time with mean rate *v* until a threshold is reached, which triggers the corresponding response (at the time labeled *response onset*). Response time is the time it takes to reach a threshold (decision time) plus an intercept term representing non-decision processes (e.g., perceptual encoding and motor response time). Note that other linking functions between drift rate and choice utility are possible. For an introduction to applying such models to experimental data, Voss et al. (2013).

Crucially, sequential sampling models can be thought of as generalizing soft-max into the time domain, which allows them to simultaneously explain how choices *and response times* arise from a common set of latent cognitive processes. This provides closer contact between model and data, additional constraint on theory, and more robust inferences. As noted in prior work (Tuerlinckx and De Boeck, 2005; Bogacz et al., 2006; Miletić et al., 2020), soft-max (Equation 2) is formally equivalent to the following sequential sampling choice function, which describes the probability that evidence first reaches the upper threshold (corresponding to an “exert effort” response in Figure 1) as:

(3) $$\begin{array}{r} {p\left(effort\right)}_{t}=\frac{1}{1+e^{-\frac{2v_{t}A}{s^{2}}}} \end{array}$$

where *v* is the mean rate of information processing (drift rate), *A* is the response threshold, *s* is the standard deviation of the evidence accumulation process, and *t* indexes trials. Equating drift rate with choice utility^2^ (Equation 1) and substituting β for *2A/s*^2^ yields the original soft-max choice function used by Chen et al. (Equation 2). Integrating Chen's models with Equation 3 would thus yield a standard sequential sampling model with choice utility-based drift rates and an additional effort-discounting parameter. Sequential sampling models explain behavior in terms of psychologically interpretable parameters (e.g., information processing speed, response caution, motor response/encoding time, choice bias), so a combined model would simultaneously decompose observed decision-making behavior into component cognitive processes and quantify effort discounting within a single theoretical framework (for a similar approach to decomposing loss aversion in risk-based decision making using a standard sequential sampling model, Clay et al., 2017).

Such an approach would offer clear benefits for the field of effort-based decision making, improving measurement and facilitating theory development by providing a more detailed characterization of group and individual differences in loss aversion. In particular, this approach could expand on Chen et al.'s results by explaining the relationship between effort discounting and additional Parkinson's-related cognitive and motor deficits known to affect response latency (e.g., longer motor response times, less efficient information processing, impaired cognitive control over thresholds, O'Callaghan et al., 2017; Servant et al., 2018). Patients prone to effort discounting may have difficulty integrating information about effort into the decision process (a drift rate effect) or set more impulsive evidence criteria (a threshold effect) compared with healthy controls. Testing these competing accounts would further our understanding of Parkinson's disease and better titrate individual differences in cognitive and motor processes. Explicit mechanisms to capture additional phenomena relevant to effort-based decision making, such as learning, urgency, and fatigue effects, can also be instantiated within the same sequential sampling framework (Milosavljevic et al., 2010; Miletić et al., 2020), and linking cognitive processes with neurophysiological measures (e.g., by treating model parameters as covariates to neural activity or constructing a joint model, Turner et al., 2017, 2019) would be especially informative regarding Chen et al.'s broader questions about the role of dopamine and Basal Ganglia activity in explaining Parkinson's-related differences in decision making. Overall, moving toward a cognitive process model of effort-based decision making promises a finer-grained mechanistic understanding of aberrant motivational states in neuropsychiatric disorders and more detailed insight into the sources of group and individual differences in effort-based decision making.

## Author Contributions

The author confirms being the sole contributor of this work and has approved it for publication.

### Conflict of Interest

The author declares that the research was conducted in the absence of any commercial or financial relationships that could be construed as a potential conflict of interest.

## Footnotes

^1^In the present context, evidence would constitute information about the relative value or utility of exerting effort vs. skipping the trial. This evidence is assumed to be sampled from noisy internal representations, in line with applications of these models to memory- and value-based decisions (e.g., Ratcliff, 1978; Krajbich and Rangel, 2011; Westbrook et al., 2020).

^2^The connection between drift rate and choice utility or value has been well-established in recent work merging reinforcement learning with sequential sampling models in the domain of value-based decision making (for a review, Miletić et al., 2020). Here drift rate is simply set equal to choice utility, however, many other linking functions are possible.

## References

[B1] Argyelan M. Herzallah M. Sako W. DeLucia I. Sarpal D.. (2018). Dopamine modulates striatal response to reward and punishment in patients with Parkinson's disease: a pharmacological challenge fMRI study. NeuroReport 29, 532–540. 10.1097/WNR.0000000000000970 29432300 PMC5895508

[B2] Baraduc P. Thobois S. Gan J. Broussolle E. Desmurget M. (2013). A common optimization principle for motor execution in healthy subjects and Parkinsonian patients. J. Neurosci. 33, 665–677. 10.1523/jneurosci.1482-12.2013 23303945 PMC6704928

[B3] Bogacz R. Brown E. Moehlis J. Holmes P. Cohen J. D. (2006). The physics of optimal decision making: a formal analysis of models of performance in two-alternative forced-choice tasks. Psychol. Rev. 113, 700–765. 10.1037/0033-295X.113.4.700 17014301

[B4] Brown S. D. Heathcote A. (2008). The simplest complete model of choice response time: linear ballistic accumulation. Cognit. Psychol. 57, 153–178. 10.1016/j.cogpsych.2007.12.002 18243170

[B5] Chen X. Voets S. Jenkinson N. Galea J. M. (2019). Dopamine-dependent loss aversion during effort-based decision-making. J. Neurosci. 40, 661–670. 10.1523/JNEUROSCI.1760-19.2019 31727795 PMC6961986

[B6] Chong T. J. Bonnelle V. Manohar S. Veromann K. R. Muhammed K.. (2015). Dopamine enhances willingness to exert effort for reward in Parkinson's disease. Cortex 69, 40–46. 10.1016/j.cortex.2015.04.003 25967086 PMC4533227

[B7] Clay S. N. Clithero J. A. Harris A. M. Reed C. L. (2017). Loss aversion reflects information accumulation, not bias: a drift-diffusion model study. Front. Psychol. 8:1708 10.3389/fpsyg.2017.01708 29066987 PMC5641396

[B8] Cléry-Melin M. L. Schmidt L. Lafargue G. Baup N. Fossati P.. (2011). Why don't you try harder? An investigation of effort production in major depression. PLoS ONE 6:e23178. 10.1371/journal.pone.0023178 21853083 PMC3154289

[B9] Forstmann B. U. Ratcliff R. Wagenmakers E.-J. (2016). Sequential sampling models in cognitive neuroscience: advantages, applications, and extensions. Ann. Rev. Psychol. 67, 641–666. 10.1146/annurev-psych-122414-033645 26393872 PMC5112760

[B10] Frank M. J. (2005). Dynamic dopamine modulation in the basal ganglia: a neurocomputational account of cognitive deficits in medicated and nonmedicated Parkinsonism. J. Cognit. Neurosci. 17, 51–72. 10.1162/0898929052880093 15701239

[B11] Frank M. J. Seeberger L. C. O'Reilly R. C. (2004). By carrot or by stick: cognitive reinforcement learning in parkinsonism. Science 306, 1940–1943. 10.1126/science.1102941 15528409

[B12] Hartmann M. N. Hager O. M. Tobler P. N. Kaiser S. (2013). Parabolic discounting of monetary rewards by physical effort. Behav. Process. 100, 192–196. 10.1016/j.beproc.2013.09.014 24140077

[B13] Kahneman D. Tversky A. (1979). Prospect theory: an analysis of decision under risk. Econometrica 47, 263–291. 10.2307/1914185

[B14] Kobayakawa M. Tsuruya N. Kawamura M. (2010). Sensitivity to reward and punishment in Parkinson's disease: an analysis of behavioral patterns using a modified version of the Iowa gambling task. Parkinsonism Related Disord. 16, 453–457. 10.1016/j.parkreldis.2010.04.011 20493754

[B15] Krajbich I. Rangel A. (2011). Multialternative drift-diffusion model predicts the relationship between visual fixations and choice in value-based decisions. Proc. Natl. Acad. Sci. U.S.A. 108, 13852–13857. 10.1073/pnas.1101328108 21808009 PMC3158210

[B16] Kurniawan I. T. Guitart-Masip M. Dolan R. J. (2011). Dopamine and effort-based decision making. Front. Neurosci. 5:81. 10.3389/fnins.2011.00081 21734862 PMC3122071

[B17] Miletić S. Boag R. J. Forstmann B. U. (2020). Mutual benefits: combining reinforcement learning with sequential sampling models. Neuropsychologia 136:107261. 10.1016/j.neuropsychologia.2019.107261 31733237

[B18] Milosavljevic M. Malmaud J. Huth A. Kock C. Rangel A. (2010). The drift diffusion model can account for the accuracy and reaction time of value-based choices under high and low time pressure. Judgment Dec. Mak. 5, 437–449. 10.2139/ssrn.1901533

[B19] Mulder M. J. Van Maanen L. Forstmann B. U. (2014). Perceptual decision neurosciences - A model-based review. Neuroscience 277, 872–884. 10.1016/j.neuroscience.2014.07.031 25080159

[B20] O'Callaghan C. Hall J. M. Tomassini A. Muller A. J. Walpola I. C. Moustafa A. A.. (2017). Visual hallucinations are characterized by impaired sensory evidence accumulation: insights from hierarchical drift diffusion modeling in Parkinson's disease. Biol. Psychiatry Cognit. Neurosci. Neuroimaging 2, 680–688. 10.1016/j.bpsc.2017.04.007 29560902

[B21] Rahman S. Sahakian B. J. Cardinal R. N. Rogers R. D. Robbins T. W. (2001). Decision making and neuropsychiatry. Trends Cognit. Sci. 5, 271–277. 10.1016/s1364-6613(00)01650-8 11390298

[B22] Ratcliff R. (1978). A theory of memory retrieval. Psychol. Rev. 85, 59–108. 10.1037/0033-295X.85.2.59

[B23] Servant M. van Wouwe N. Wylie S. A. Logan G. D. (2018). A model-based quantification of action control deficits in Parkinson's disease. Neuropsychologia 111, 26–35. 10.1016/j.neuropsychologia.2018.01.014 29360609 PMC5916758

[B24] Treadway M. T. Bossaller N. A. Shelton R. C. Zald D. H. (2012). Effort-based decision-making in major depressive disorder: a translational model of motivational anhedonia. J. Abnormal Psychol. 121, 553–558. 10.1037/a0028813 22775583 PMC3730492

[B25] Tuerlinckx F. De Boeck P. (2005). Two interpretations of the discrimination parameter. Psychometrika 70, 629–650. 10.1007/s11336-000-0810-3

[B26] Turner B. M. Forstmann B. U. Love B. C. Palmeri T. J. Van Maanen L. (2017). Approaches to analysis in model-based cognitive neuroscience. J. Math. Psychol. 76, 65–79. 10.1016/j.jmp.2016.01.001 31745373 PMC6863443

[B27] Turner B. M. Forstmann B. U. Steyvers M. (2019). Joint Models of Neural and Behavioral Data: Computational Approaches to Cognition and Perception. Cham: Springer International Publishing 10.1007/978-3-030-03688-1

[B28] Tversky A. Kahneman D. (1992). Advances in prospect theory: cumulative representation of uncertainty. J. Risk Uncertainty 5, 297–323. 10.1007/978-3-319-20451-2_24

[B29] Voss A. Nagler M. Lerche V. (2013). Diffusion models in experimental psychology: a practical introduction. Exp. Psychol. 60, 385–402. 10.1027/1618-3169/a000218 23895923

[B30] Westbrook A. van den Bosch R. Määttä J. I. Hofmans L. Papadopetraki D. Cools R.. (2020). Dopamine promotes cognitive effort by biasing the benefits versus costs of cognitive work. Science 367, 1362–1366. 10.1126/science.aaz5891 32193325 PMC7430502

